# Population health management within the primary care context – scoping review

**DOI:** 10.1080/13814788.2026.2633852

**Published:** 2026-03-09

**Authors:** Emmily Schaubroeck, Giorgio Sessa, Jan De Maeseneer, Sara Willems, Peter Decat

**Affiliations:** ^a^ Department of Public Health and Primary Care, Ghent University, Ghent, Belgium; ^b^ Department of Life Sciences and Public Health, Università Cattolica del Sacro Cuore, Rome, Italy

**Keywords:** Population health management, primary care, general practice, conceptualisation

## Abstract

**Background and objectives:**

Population health management (PHM) is increasingly promoted as a strategy to improve health outcomes, enhance healthcare quality, reduce costs, and, more recently, support clinician well-being and advance health equity – the Quintuple Aim. However, how PHM is conceptualised within the primary care context remains unclear. This scoping review explores how PHM is conceptualised within this context.

**Method:**

Five databases (PubMed, Embase, CINAHL, Web of Science and Scopus) were searched to find publications that conceptually addressed PHM and its interaction with the primary care context. Data extraction focused on definitions and related terms, the bidirectional influence between PHM and general practice, and interpretations of the components ‘population’ and ‘management’.

**Results:**

27 publications were included. Definitions of PHM varied, with few explicitly addressing the primary care practice level. They highlighted the need to proactively address social determinants of health beyond clinical outcomes. Both top-down and bottom-up dynamics make general practices accountable for and increasingly involved in the identification of populations, risk stratification and impact assessment, with both clinical judgement and real-world primary care data. Management involves team-based and technology-supported care.

**Conclusion:**

Considering PHM within primary care highlighted the importance of general practice’s accountability, its consideration of social determinants of health beyond clinical outcomes and its community alignment to enhance equity. What the potential added value of general practitioner’s clinical intuition and real-world primary care data in assessing impact warrants additional exploration.

## Introduction

Population Health Management (PHM) is widely regarded as an approach to achieve population health [1]. An international analysis, structuring PHM activities according to their contribution to the Triple Aim in healthcare [2] – improve population health, improve the care experience and lower costs – pointed out that PHM’s overarching goals, activities and contextual factors are often not clearly described [3]. Furthermore, PHM initiatives tend to focus on system-level activities, with less emphasis on clinical and professional integration [4], although clinician well-being is increasingly getting attention as it was added as a fourth to the Triple Aim, resulting in the Quadruple Aim [5].

In 2023 the WHO European region defined PHM as ‘a data-driven, people-centred and proactive approach to managing the health and well-being of a defined population that can enable primary healthcare providers to move to targeted and tailored interventions that account for the needs of different groups and individuals’ [6]. It helps them in adopting a holistic and proportionate universalism approach to address health inequalities at the community level [6], which resonates the fifth of the Quintuple Aim – advance health equity [7]. Despite these expectations of primary care providers there’s a gap in understanding how PHM is applied [8], not in the least within the general practice context of primary care (further primary care) [9,10].

Therefore, this scoping review addresses the following research question: How is population health management being conceptualised within primary care?

## Method

As the goal is to inform practice with a comprehensive understanding [11] of PHM within primary care, the scoping review method was chosen over a concept analysis [12].

## Literature search

Search strings were created for five databases (PubMed, Embase, CINAHL, Web of Science and Scopus) and optimised by The Knowledge Centre for Health Ghent (KCGG). Free-text terms related to ‘population health’ and ‘management’ were refined through an iterative search process. Wildcard, truncation and proximity operators were used to explore various word combinations and ‘strategy’ and ‘approach’ as alternatives to ‘management’. While terms like ‘assess’, ‘measure’ or ‘improve’ were identified as related, they were excluded to avoid restricting the concept to data-specific subtopics. No restrictions in publication date were set. The search was last reconducted on 28 April 2025. E.g. the Pubmed via Ovid search string:

**Table:** 

(exp Population Health Management/) OR (((population adj2 (manag* or (health strateg* or health approach*))) or ‘population medicine’).ti,ab,kf.) AND ((exp primary health care/or exp physicians, primary care/or exp family practice/or exp physicians, family/or exp general practice/or exp general practitioners/or exp ambulatory care/) OR (‘primary health care’ or ‘primary care’ or ‘primary medical care’ or ‘family practi*’ or ‘family medicine’ or ‘family physician*’ or ‘general practi*’ or gp or gps or ‘ambulatory care’ or ‘outpatient*’).ti,ab,kf.)

### Study selection and data extraction

**Figure 1.**
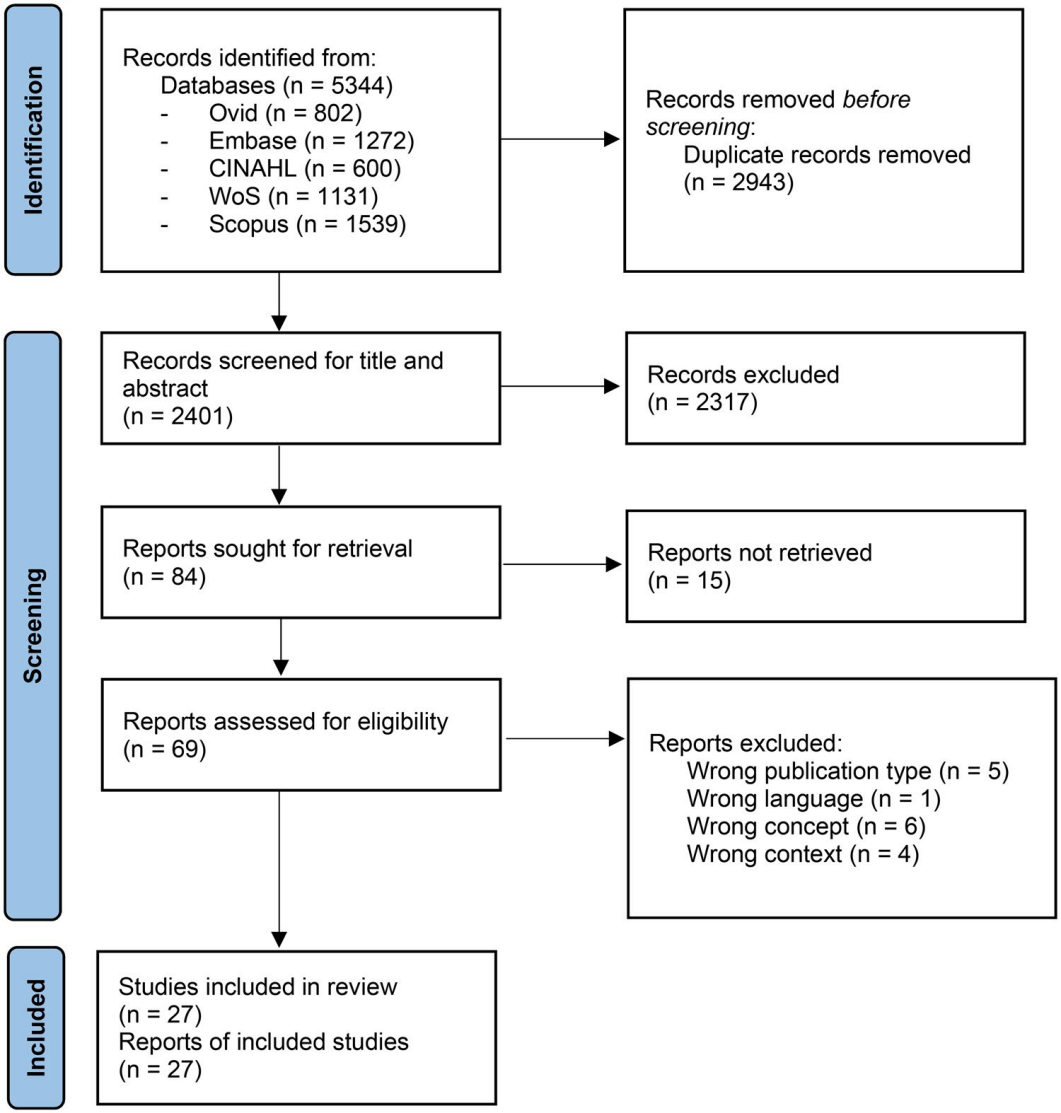
PRISMA flow chart.

To ensure inclusion of relevant content even if the exact term ‘PHM’ was not explicitly used, artificial intelligence tools were not used to screen articles. Articles in English, German, French, Dutch, Italian, Spanish and Portuguese were included if they had an available English abstract. The PRISMA-ScR guidelines were followed for reporting (Figure 1) [13].

The inclusion criteria followed the PCC-method (Participants, Concept, Context). As the study focused on conceptualising ‘population’ in PHM, no restrictions were placed on study participants. The context was limited to the general practice within primary care, defined in this paper as the organisational entity delivering primary healthcare as outlined by WHO [14]. It is considered the micro-level of the healthcare system [15], traditionally where providers address individual patient concerns in Western countries [16] and is closely related to the meso-level (organisations like hospitals) and macro-level (governmental health policy) of healthcare systems. No specific study types were excluded. Conference abstracts and grey literature were not included.

Titles and abstracts were screened independently by two reviewers. Full-texts were assessed based on inclusion criteria with inter-operator validity checked. After the first reading, data extraction was discussed with a third researcher, resulting in the following extraction questions: (1) What definitions and related terms exist?; (2) How does the primary care context influence PHM, and vice versa?; (3) How is the population component conceptualised?; and (4) How is the management component conceptualised?

## Results

This scoping review identified 27 publications that conceptually addressed the phenomenon PHM (including the ones using other terms) and considered its relationship to the primary care context (Table 1). Most of the included publications focused on the U.S. healthcare system (*n* = 15), while others originated from Canada (*n* = 3), the UK (*n* = 3), Australia (*n* = 2), the Netherlands (*n* = 1) and Spain (*n* = 1) and the European part of the WHO (*n* = 1).

**Table 1.** Selected articles.

Author(s)	Country	Year	Title
Bazemore, A. [31]	USA	2010	Harnessing geographic information systems (GIS) to enable community-oriented primary care
Bearden, T. [40]	USA (about Ghana, Mongolia)	2019	Empanelment: A foundational component of primary health care
Cerezo-Cerezo, J. [18]	Spain (WHO Europe)	2023	Unlocking the power of population health management to strengthen primary health care
Coe, A. B. [25]	USA	2017	Pharmacists supporting population health in patient-centred medical homes
Davies, D. [34]	Australia	2020	Primary Sense: a new population health management tool for general practice
Edwards, I. [44]	Australia UK	2008	Clinical reasoning and population health: Decision making for an emerging paradigm of health care
Farmanova, E. [27]	Canada	2019	Combining Integration of Care and a Population Health Approach: A Scoping Review of Redesign Strategies and Interventions, and their Impact
Girwar, S. A. M. [37]	Netherlands	2021	A systematic review of risk stratification tools internationally used in primary care settings
Gofin, J. [26]	USA	2015	Community-oriented primary care (COPC) and the affordable care act: an opportunity to meet the demands of an evolving health care system
Grundy, P. H. [23]	USA	2015	Health IT’s essential role in the patient-centred medical home and practice-based population health management
Haas, S. A. [24]	USA	2016	Developing Staffing Models to Support Population Health Management And Quality Outcomes in Ambulatory Care Settings
Hobson, A. [45]	USA	2017	Improving the care of veterans: The role of nurse practitioners in team-based population health management
Johnson, T. L. [41]	USA	2015	Augmenting Predictive Modelling Tools with Clinical Insights for Care Coordination Program Design and Implementation
Li, Y. [32]	USA	2014	Using systems science for population health management in primary care
Maher, K. [30]	USA	1997	Identifying opportunities to improve the management of care: A population-based diagnostic methodology
Mant, D. [36]	UK	1985	Community general practitioner
McShane, M. [35]	UK	2020	Making it personal – population health management and the NHS
Mohanty, N. [43]	USA	2021	The People, Process, and Technology for Population Management in Community Health
North, F. [39]	USA	2018	Population health challenges in primary care: What are the unfinished tasks and who should do them?
Nuño-Solinís, R. [20]	Spain	2012	An answer to chronicity in the Basque Country: Primary care-based population health management
Orlowski, A. [38]	UK	2021	Bridging the impactibility gap in population health management: A systematic review
Ottmar, J. [22]	USA	2015	Family Physicians’ Ability to Perform Population Management Is Associated with Adoption of Other Aspects of the Patient-Centred Medical Home
Reddy, A. [28]	USA	2015	Risk stratification methods and provision of care management services in comprehensive primary care initiative practices
Rouble, A. N. [9]	Canada	2019	Integrating clinical medicine and population health: where to from here?
Tishler, J. [21]	USA	2020	Quality improvement and population management in adult primary care
Vaghefi, I. [29]	Canada USA	2016	Understanding the Impact of Electronic Medical Record Use on Practice-Based Population Health Management: A Mixed-Method Study
Wagner, E. H. [33]	USA	2018	How do innovative primary care practices achieve the quadruple aim?

### Definition/description of the concept and related terms

Fourteen of the selected articles defined or described PHM within a primary care context, of which six mentioned concepts related to PHM in the context of primary care. The overview can be found in Table 2.

**Table 2.** The concept of population health management (PHM)and related terms (14 studies).

Definition/description of PHM within PC practice context (*n* = 8)	
**- Referring to the most used definition of population health as** an approach to improve the health outcomes of a group of individuals, including the distribution of outcomes within the group [17] **(*n* = 4)** [23,25,43,45] **- Referring to practice-based population health (PBPH) management as** an approach to care that uses information on a group (‘population’) of patients within a primary care practice or group of practices (‘practice-based’) to improve the care and clinical outcomes of patients within that practice [19] **(*n* = 2)** [23,29] - **Primary care-based population health management** makes it possible to analyse the complexity and comorbidity levels of the people and facilitates a segmentation of the population to plan and distribute resources in such a way that the needs of the different groups of citizens are met in a customised and proactive manner [20] - **Population health management (PHM)** as a people-centred, data-driven and proactive approach to improving the health and well-being of a defined population, considering the differences within that population and their social determinants of health [18]	
Related terms (*n* = 6)	
Total health management [30]	A comprehensive, population-based approach to managing health care risk, access and care; modifying clinical decision-making processes in order to eliminate variation in and to rationalise resources thereby enabling breakthrough reductions in utilisation and medical costs while improving or maintaining quality
Community-Oriented Primary Care (COPC) [26]	Is focused on providing care to a defined population, based on their assessed needs, to improve health status combining primary care and population health
Community general practitioner [36]	Combines clinical skills with the skills of population medicine
Clinical population medicine [9]	The integration of population health into clinical practice [46]
(Population) panel management [45]	Operationally defined as a team-based healthcare delivery model designed to identify and engage patients in preventive care services and chronic disease management [48]
Empanelment [40]	Continuous, iterative set of processes that identify and assign populations to facilities, care teams, or providers who have a responsibility to know their assigned population and to proactively deliver coordinated primary health care towards achieving universal health coverage

Some articles referred to Kindig’s general definition of population health [17] and the most recent one discussed the 2023 PHM definition of WHO European region [18]. Two cited a definition emphasising clinical outcomes in patients [19] and one focused on addressing the needs of people – not patients – with complexity or comorbidity [20].

### How the primary care context is influenced by or influencing the phenomenon of PHM

On the one hand, examples were found of general practices directly affected by higher level decisions in a top-down way. On the other hand, the primary care context is driving grassroots initiatives installing PHM in a bottom-up way.

Top-down approaches are primarily policy-driven, with macro-level forces, such as national reforms in the U.S. [21–26]. Aiming to reorganise healthcare systems [24] and reform payment models [27] to improve the care experience, the second of the Triple Aim. Risk-stratified care management is seen as vital for improving population health [28], the first Aim. PHM helps to reduce healthcare expenses [29] – the third Aim – by identifying high-cost populations early [30] and tackling workforce shortages on the primary care level [31], which resonates the fourth Aim of clinician well-being.

These efforts cascade to the meso level, where organisations aim to optimise care coordination over time and across healthcare providers [23], and to the micro level, where individual general practices align with the Triple Aim [32]. For example in the US, Primary Care Medical Homes (PCMHs) were developed to focus on PHM [21,22,33]. At this level, general practitioners are held accountable for all care [21,23] for a total population [9,21,23,30].

In countries like Australia and the UK, meso-level primary care networks (PCNs) were created for timely and aggregated patient data to track outcomes [34] and as central to PHM in an integrated care system [35] to address unmet needs, especially in disadvantaged communities. As a consequence, the micro-level general practices experienced a responsibility for accurate individual-level data [34].

Conversely, bottom-up approaches were found. The oldest selected article introduced the *community general practitioner* and argued that quality in general practice requires acknowledging the public health content in primary care [36]. Clinical population medicine [9] or management [31] integrates population health into clinical practice. More recent publications also highlight the role of primary care in reducing costs, improving outcomes and addressing health inequalities, especially through chronic disease management [18,20] and addressing social determinants of health [27]. Furthermore, effective use of real-world primary care data for risk stratification [18,37] and predictive analysis [38], along with an emphasis on prevention [39] and outreach [21] seems key to extending integrated care benefits to the general population [27]. The most recent article [18] confirmed that primary care traits allow strengthening PHM by drawing attention to the social determinants of health. It concluded that the relationship between primary health care and PHM is that of a virtuous circle as PHM helps primary care providers identify populations to target and PHM provides a pragmatic framework for providers to operationalise a number of essential public health functions.

### The ‘population’ component

Considering PHM within the context of primary care brings up particularities concerning the component ‘population’ listed in Table 3. Accountability is central, with calls for proactive recruitment [40,41] and explicitly defining [21,41] and registering [36] the accountable population through empanelment [40], where care is delivered to all who could benefit.

**Table 3.** The population component.

Population component in the primary care context (*n* = 2)
**Accountability for a population** 1. Practice attending population [34] versus proactive [40,41] - geography-based accountability [31,35] - insurance-based accountability [40,41] 2. Dynamic in time 3. Need for explicitly defining [9,41] 4. Registration in practice [26] in patient panels [26,36,40] **Application of the PHM-cycle** [18] **1) Identification of a target population** Entire population versus a subset based on: geography [9,44] demographics [44] health needs [18], their determinants and assets in community [26] a health problem/condition/diagnosis [22,29,44] risk exposure [18,29] uncontrolled health problems [33] or gaps in care [25,32,39,45] needing outreach [21] who could benefit from primary care [41] Dynamic in time: yearly [20,25,40] versus monthly [41] **2) Segmentation based on:** demographics [35,37] (primary) health care needs [27,28] clinical intuition [28] clinical factors: diseases [27,37], morbidity [37] or complexity [27,35] risk profile [27] healthcare costs [27,30] healthcare utilisation patterns/claims [37] e.g. missed recent or routine care [43] **3) Risk stratification** Need for explicitly risk stratifying an accountable population [41] Based on: demographic/social information [20] risk/needs profiles [27] clinical information: e.g. pharmacological [20] risk for adverse event [37] extent of healthcare utilisation [20,37] **4) Impactibility** [38] Prioritisation of patients most likely to respond to care AND whose treatment would maximise the likelihood of achieving the Triple Aim [38] Based on: provider ‘check’ [24,35,38] versus objective data (claims data, quality data, healthcare utilisation data) [24]

The general PHM-cycle by Struijs et al. [42] – comprising identification, segmentation, stratification, intervention, and impact assessment – is also being applied within this micro-level context [18]. The first step is identifying a target population, either broad or a subset based on different criteria. The next step is segmentation to distribute resources to meet the customised needs of different groups [20]. The criteria for identification and segmentation overlap. Lastly, risk stratification divides the segmented population into tiers [28], using both objective data [24] and clinical judgement [38] to select those most likely to benefit from care [38]. This leads to the last step of the circle, namely checking the impactibility [38].

### The ‘management’ component

The management component of PHM shows particularities in terms of content, characteristics and the process of development that requires people and technology, shown in Table 4.

**Table 4.** The management component.

Management component in the primary care context (*n* = 25)
**1) Content:** 1) Both population and individual level: from a combination (considering the needs [9,36], carrying out initiatives [27] and managing care [30] on both levels) to an integration (community-based management of social determinants of health [9,43] or chronic conditions [44]) 2) Risk/disease management across the care continuum [18] (prevention/health promotion [29,40,45] and chronic disease management [45]) 3) Need for outreach [22,25] **2) Characteristics:** Proactive [18,20,32,40] Patient-driven [45] or patient-centred [41] Personalised [18,45] Team-based [23,45] Integrated [20,23] Technology-based [20] Holistic [18] **3) Process:** 1) Changing accountability for population health outcomes: providing comprehensive ambulatory care services [41] and providers accountable [21] 2) Reorganisation: from developing [20] or expanding the capacity of general practices [25] to redesigning primary care practice to develop capacity [27] e.g. *community-oriented primary care (COPC)* [26] 3) Analysis of practice data to identify gaps in care [32,45] **4) People:** 1) Stable relationships between patients and physician-directed integrated team [23] 2) Staffing according to (patient population’s) need [24] and broadened [23] 3) Specific training [24,45]: e.g. staff with data analysis skills [33] 4) Task shift: e.g. nurse practitioners handling as primary care providers [45] **5) Technology:** 1) Data: - From all levels of the healthcare system [35] into predictive modelling of disease progression [32,41] - Assessing the whole population using routinely collected primary care data [37]- integration of clinical data with population data [31] - Recognition of the range of knowledge sources that are required for data gathering and subsequent decision making in clinical practice [44] 2) Asynchronously from clinical encounter [43] versus during face to face visits [39] aligned to workflow [34] 3) Incentivise qualified EHR technology [21]

It involves efforts at both individual and population level [9,27,30,36]. An integrative approach suggests community-based management of social determinants of health [9,43] or individual chronic disease [44]. Management activities include preventive care and health promotion [29,40,45], and chronic disease management [45] along the care continuum [18]. This implies outreach [22,25].

The management is characterised by proactive [18,20,32,40], patient-centred [41], personalised [18,45], team-based [23,45], integrated [20,23], technology-supported [20] and holistic [18] care. Processes involve shifting accountability [41], reorganisation [20] and identification of care gaps [32,45].

Human resources are vital, with a focus on stable patient – provider relationships [23], need-based staffing [24], specialised training [24,45] (e.g. in data analysis [33]), and task shifting to roles like nurse practitioners [45]. Technology plays a key role by enabling data collection across levels of care [35] into predictive modelling [32,41]. Examples were the use of primary care data to assess the whole population [37], integration of clinical with population data [31] and data guiding decision making [44]. Technology should support asynchronous [43] and real-time [34,39] decision-making, and promote the use of certified electronic health record systems [21].

## Discussion

This scoping review offers a comprehensive synthesis of how PHM is conceptualised and operationalised within general practice across diverse health systems. The findings reveal both heterogeneity and convergence in how PHM is understood, adapted, and integrated into general practice, highlighting its dual role as both a product of broader health policy reforms and a driver of grassroots general practice initiatives.

### The concept of PHM

The definitional landscape of PHM within primary care is marked by conceptual multiplicity. Only a minority of sources directly addressed PHM as it manifests at the primary care level, focusing either on clinical outcomes or people’s needs. Focusing solely on clinical outcomes risks overlooking populations who rarely engage with the healthcare system and thus are not registered with a clinical problem, which does not necessarily mean they are free of health issues. ‘(Population) panel management’ assumes all citizens are registered in PC practices, which remains untrue in many countries. Future policy efforts should prioritise accountability for a clearly defined population, in order to address people’s needs beyond conventional clinical outcomes. An international analysis of Triple Aim–related measures for PHM highlighted the dominance of health outcomes over the participation domain, as well as the underlying conservative conception of health as a deficit [3]. The WHO European Region’s articulation of PHM adopted the proactivity observed in recent literature, underscoring equity and social determinants of health as core tenets. Proactivity requires continuous decisions on which health determinants to focus, as addressing societal needs often extends beyond the healthcare system’s reach, let alone beyond general practice’s reach.

The retrieved related concepts further indicate the multidimensional evolution of PHM and confirm the discordance between focusing on clinical outcomes versus on people’s – not necessarily patient’s – needs. On the one hand, ‘total health management’ asks for the modification of clinical decision-making processes beyond traditional chronic disease management to improve equity and rationalise resources. On the other hand, the adaptation of primary care to address population needs, known as ‘community oriented primary care (COPC)’, merges primary care with population health. A commentary article highlighted inconsistencies in ‘clinical population medicine’ [46], also ranging from incorporating the social determinants of health into clinical practice to broader community health planning, emphasising the need to complement public health efforts. Clarifying the relationship between these concepts for policy makers could be helpful.

### The context of primary care

The interaction between PHM and primary care is characterised by a dynamic, bidirectional influence that transcends traditional top-down policy implementation models. Top-down pathways were observed in the U.S., where macro-level initiatives as PCMHs have translated the Triple Aim into organisational mandates that recalibrate PC delivery. Notably, these models position general practitioners as accountable for population-level outcomes. It is questionable whether this will enhance equity rather than induce cherry-picking of the easiest population to treat.

Conversely, bottom-up innovation illustrates how general practices themselves can incubate and scale PHM-aligned approaches. Though digitalisation has shifted monitoring and planning to higher system levels, the most recent article stated PHM helps providers to operationalise essential public health functions. As noted by van Ede et al. it remains essential to involve providers in reporting on PHM initiatives, with particular attention to their clinical and professional integration, in order to elucidate the contribution of general practices [4]. Data-informed, proactive care is seen as a practice-level contribution to population health goals. It remains unclear whether the alignment and responsiveness of health care organisations to the community within which they reside generally is enough to effectively enhance equity, let alone balance all five of the Quintuple Aim. Future research investigating this alignment could be helpful to inform policy.

Whereas in the top-down perspective the meso-level was seen as an in between-level also striving for the Triple Aim, there were systems in which the meso-level was created more bottom-up by gathering general practices into Primary Care Networks (PCNs) to address local community needs. It remains uncertain how this helps general practice in doing so and this could be interesting to investigate.

### The ‘population’ in PHM within primary care context

The population component of PHM is translated from an abstract aggregate to a concretely defined and managed group on the primary care practice level. Accountability reconfigures the primary care team’s responsibility beyond those who seek care to those who might benefit from care. This is in line with the findings of Steenkamer et al.’s review on PHM claiming that local action across settings and stakeholders to address the full range of health determinants requires accountability mechanisms [8].

While identification, segmentation, stratification, intervention, and impact assessment are often conceived as meso- or macro-level responsibilities, the review showed how general practices are increasingly engaging in them, with both objective real-world primary care data and subjective clinical judgement informing care prioritisation, focusing limited resources on patients most likely to benefit from proactive care. In primary care systems lacking fixed patient populations, identifying patients who could benefit from primary care and registering them in practices can be the main PHM strategy. Apart from incomplete primary care data, the general cross-sectional way to identify patients rather than considering patients by iterative follow-up limits effective impact modelling [47]. A future focus on longitudinal tracking of the accountable population could provide better data for research.

### The ‘management’ in PHM within the primary care context

Management changes reflect shifting accountability, raising content challenges for PC providers in balancing individual care with population needs. Similar to holding PC providers accountable for clinical population health outcomes, holding them accountable for healthcare utilisation may also incentivise patient selection and risks overlooking social determinants of health.

The characteristics of PHM-aligned management echo a shift towards proactive, team-based, technology-supported care. Importantly, these approaches are not only technical but relational, relying heavily on stable patient-provider relationships, interdisciplinary teamwork, and skill development. Task shifting is both a necessity in the context of workforce shortages and a strategic alignment with the Fourth Aim, which addresses provider well-being and sustainability. It remains uncertain whether subjective clinical judgement is valued to the same extent as objective real-world patient data within algorithmic models for guiding care management aimed at addressing unmet needs amenable to timely intervention. This would be a valuable focus for future research.

In summary, these considerations regarding population and management underscore the pivotal role of primary care across several of the six PHM elements identified by Van Ede et al. particularly the accountable regional organisation, integrated data infrastructure, and the co-design of workforce and community [4]. Moreover, the importance of a shared organisational vision within PHM – especially in achieving an appropriate balance between the interdependent aims of the Quintuple Aim, as emphasised by Struijs et al. – appears to be relevant also in the context of primary care [42].

### Strengths and limitations

This review is the first to focus on PHM within the primary care context but is limited by the semantic confusion surrounding the term PHM and by limited comparability in the role and positioning of general practice across healthcare systems. However, the dominant U.S. articles themselves rendered a broad scope of characteristics crucial to discussing the PHM concept within this context and showed that any generalisation requires careful contextualisation. The review process involved collaboration between two reviewers from different European countries, ensuring a diverse perspective. Any incongruence was discussed with a third reviewer. The presentation of the results in a descriptive format answering the sub-questions of the review generated an overview of the particularities of the concept PHM within this specific primary care context. Although examining such an extensive concept within this relatively narrow context did not substantially enhance conceptual clarity, this focused overview of context-specific characteristics facilitated the identification of research gaps and may inform practice.

### Implications for policy and future research

This review showed that the context of primary care matters for PHM to fulfil its potential. Clarifying its relationship to related concepts as public health and COPC could be helpful for both policy makers and researchers to understand and foster bottom-up and top-down integration of existing PHM implementations. PHM’s relevance and legitimacy rest on its ability to respond to the lived realities of PC providers and the populations they account for. Future policy efforts should prioritise this accountability and the consideration of social determinants of health beyond clinical outcomes. Future research should envision how these social determinants of health can be addressed, whether general practice’s community alignment is sufficient to enhance equity, whether the meso-level helps primary care practices in addressing unmet needs and what the added value of general practitioner clinical intuition and real-world primary care data may be in impact assessment.

## Conclusion

This review shows that while PHM is a system-level ambition, primary care as a context brings along particularities in its implementation. Both total ‘population’ accountability and total care ‘management’, as higher-level components of PHM, indicate a shift in responsibilities within primary care practices. A role for primary care provider intuition in risk stratification and impactibility estimations was highlighted. Addressing social determinants of health beyond clinical outcomes and general practice’s alignment to the community in which they reside to enhance equity seem essential.
